# Third‐Generation EGFR‐TKIs in T790M‐Negative NSCLC After First/Second‐Generation EGFR‐TKI Failure: A Retrospective Study

**DOI:** 10.1002/cam4.71302

**Published:** 2025-12-16

**Authors:** Zhen Cheng, Jiali Dong, Huihao Lu, Chuhong Huang, Shujun Li, Yanming Lin, Yuting Chen, Yongcun Wang, Yanli Mo, Zhixiong Yang, Wenmei Su

**Affiliations:** ^1^ Department of Oncology Affiliated Hospital of Guangdong Medical University Zhanjiang Guangdong China; ^2^ Zhanjiang Key Laboratory of Tumor Microenvironment and Organoid Research Zhanjiang Guangdong China

**Keywords:** epidermal growth factor receptor, non‐small cell lung cancer, resistance, T790M, tyrosine kinase inhibitor

## Abstract

**Purpose:**

In non‐small cell lung cancer (NSCLC) patients resistant to first‐ or second‐generation epidermal growth factor receptor (EGFR) tyrosine kinase inhibitors (TKI), only half develop the T790M mutation and thus qualify for the treatment using third‐generation EGFR‐TKIs. For T790M‐negative patients, chemotherapy is the recommended second‐line treatment. We compared the efficacy between third‐generation EGFR‐TKIs and chemotherapy with or without first/second‐generation EGFR‐TKIs in T790M‐negative patients.

**Patients:**

This study included T790M‐negative patients with EGFR‐mutated advanced NSCLC and progressing after first‐line treatment with first‐ or second‐generation EGFR‐TKIs. Data on clinical characteristics, disease features, and survival were collected, including general conditions, medical history, imaging, histology, and molecular profiling.

**Results:**

Of 82 patients progressing after first‐ or second‐generation EGFR‐TKIs without acquiring T790M, 45 received third‐generation EGFR‐TKIs and 37 received chemotherapy and/or first/second‐generation EGFR‐TKIs. We found that third‐generation EGFR‐TKIs led to significantly longer median progression‐free survival (mPFS) than other treatments [10.20 months vs. 5.70 months, *p* = 0.017]. Subgroup analyses indicated that third‐generation EGFR‐TKIs had similar mPFS to the chemotherapy group but were significantly superior to first/second‐generation EGFR‐TKIs with or without chemotherapy (10.20 months vs. 4.80 months, *p* < 0.001; 10.20 months vs. 3.30 months, *p* = 0.004). The median overall survival (mOS) for patients treated with third‐generation EGFR‐TKIs and with non‐third‐generation therapy was 39.80 months (95% CI 23.14 to 56.46) and 32.40 months (95% CI 18.71 to 46.09), *p* = 0.408, respectively.

**Conclusions:**

Third‐generation EGFR‐TKIs significantly improved mPFS in T790M‐negative patients with EGFR‐mutated advanced NSCLC and resistant to first‐line treatment with first‐ or second‐generation EGFR‐TKIs.

**Trial Registration:**

ChiCTR2500096109

**Abbreviations**
AE: adverse event
BMI: Body mass index
CR: complete response
ECOG: Eastern Cooperative Oncology Group
EGFR: epidermal growth factor receptor
NSCLC: non‐small cell lung cancer
ORR: objective response rate
OS: overall survival
PCR: polymerase chain reaction
PD: disease progression
PFS: progression‐free survival
PR: partial response
SD: stable disease
TKI: tyrosine kinase inhibitors

## 1 Introduction

Lung cancer is one of the most common cancers and the leading cause of cancer‐related death worldwide. Activating mutations of the epidermal growth factor receptor (EGFR) gene act as an important oncogene for poor clinical outcomes and occur in about 50% of patients with lung adenocarcinoma in Asia. The most common alterations in the EGFR gene are the deletion of exon 19 and L858R point mutation in exon 21. Among these patients, 70% are sensitive to treatment with first‐ and second‐generation EGFR‐tyrosine kinase inhibitors (TKIs) [1, 2, 3].

Unfortunately, all patients treated with EGFR‐TKIs eventually develop resistance after a median of 9–15 months, due to the T790M mutation in the EGFR [4]. Second‐line systemic therapy needs to be considered when a patient develops symptomatic progression. For patients progressed with the EGFR T790M mutation after first‐ and second‐generation TKIs, Osimertinib and other third‐generation EGFR‐TKIs are recommended to overcome the acquired resistance [5, 6, 7]. For patients without the T790M mutation at the time of disease progression, platinum‐based chemotherapy rather than third‐generation EGFR‐TKIs is the standard treatment. However, it remains unclear whether the third‐generation EGFR‐TKIs show similar disease control rates or survival benefits compared to platinum‐based chemotherapy [8].

There are only a few studies that provide the survival data and analysis of third‐generation targeted drugs in T790M‐negative patients with EGFR‐mutated non‐small cell lung cancer (NSCLC) after progression on first‐ or second‐generation EGFR‐TKIs treatment. A single‐arm Phase II study (WJOG790L) has evaluated the efficacy of Osimertinib in treating T790M‐negative NSCLC patients with resistance to first/second‐generation EGFR‐TKIs [9]. As of the cutoff date of February 28, 2022, with a median follow‐up time of 13.2 months, the objective response rate (ORR) was 29.1%, the median progression‐free survival (PFS) was 4.07 months, and the median overall survival (OS) was 13.73 months [9]. The TREM study administered Osimertinib to EGFR‐mutated NSCLC patients who had failed at least one treatment with EGFR‐TKIs, regardless of their T790M mutation status. The results showed that the ORR of T790M‐negative patients treated with Osimertinib was 28%, and the PFS was 5.1 months [10]. A retrospective collection from Beijing Cancer Hospital involved 12 T790M‐negative NSCLC patients who received treatment with Almonertinib after disease progression on first‐ or second‐generation EGFR‐TKIs. The median follow‐up time was 15.6 months, with an ORR of 16.7%, a disease control rate of 83.3%, and a median progression‐free survival of 11.0 months. These findings suggest that third‐generation EGFR‐TKIs might have efficacy similar to pemetrexed chemotherapy in T790M‐negative NSCLC patients and could be a potential treatment option for T790M‐negative patients who have previously received first‐ or second‐generation EGFR‐TKIs treatment.

Here, we conducted a retrospective study to assess the efficacy and safety of third‐generation EGFR‐TKIs compared with chemotherapy in patients with T790M‐negative EGFR mutated NSCLC progressing on first‐ or second‐generation EGFR‐TKIs treatment.

## 2 Materials and Methods

### 2.1 Patients

We included advanced EGFR‐mutated NSCLC patients who were treated at the Affiliated Hospital of Guangdong Medical University between 2018 and 2024 and experienced disease progression after first‐line treatment with first‐ or second‐generation EGFR‐TKIs, with no T790M mutation at the time of progression. Imaging studies were used to confirm disease progression after treatment with first‐ or second‐generation EGFR‐TKIs. Tumor tissues were re‐collected after progression for real‐time polymerase chain reaction (PCR) analysis or next‐generation sequencing. When tumor tissues were unavailable or the patient refused to undergo another biopsy, genetic testing was conducted using circulating tumor DNA extracted from blood samples. T790M‐negative status was defined as the lack of the T790M mutation despite the presence of other activating mutations in the EGFR gene. We collected clinical characteristics and disease‐related features of patients, including general conditions, past medical history, imaging, histology, and molecular profiling.

### 2.2 Ethics Statement

This study was conducted in compliance with the principles of the Declaration of Helsinki and was approved by the Ethics Committees of the Affiliated Hospital of Guangdong Medical University (LY2024‐10‐013). All participants in the study have been given informed consent.

Registry and the Registration No. of the study: Chinese Clinical Trial Registry (ChiCTR2500096109).

### 2.3 Efficacy Assessments and Procedures

Retrospective collection of imaging data was performed, encompassing chest and abdominal CT scans, brain imaging, and bone scan data. The assessment of tumor response and progression was based on the RECIST 1.1 criteria. PFS is defined as the time from the first dose of medication to objective disease progression or death by any cause. OS is defined as the time from the first administration of medication to death by any cause. Tumor objective response evaluation is categorized based on the relief conditions assessed by RECIST 1.1 standards: CR (complete response), PR (partial response), SD (stable disease), and PD (disease progression). In the evaluation of tumor response using RECIST 1.1 criteria, the current tumor burden was compared with the smallest tumor burden observed during the study to assess whether the tumor has progressed. If there is no observed increase in the size of the target lesions compared to the prior nadir during subsequent follow‐up, it is considered that there is no PD. If an increase in tumor volume is observed, or new lesions appear, this will be defined as PD, at which point the progression will be recorded as an outcome of the follow‐up, indicating a worsening of the condition or the ineffectiveness of the treatment.

### 2.4 Statistical Analysis

Continuous data were analyzed using student's t‐test and described using the minimum value, maximum value, mean, and standard deviation. The chi‐squared test or Fisher's exact test was used for categorical data, which were described by observations and their corresponding percentages. All time‐to‐event data were calculated using the Kaplan–Meier survival analysis. All statistical descriptions and analyses were performed with SPSS Statistics, Version 27.0.

## 3 Results

### 3.1 Patient Characteristics

**FIGURE 1:**
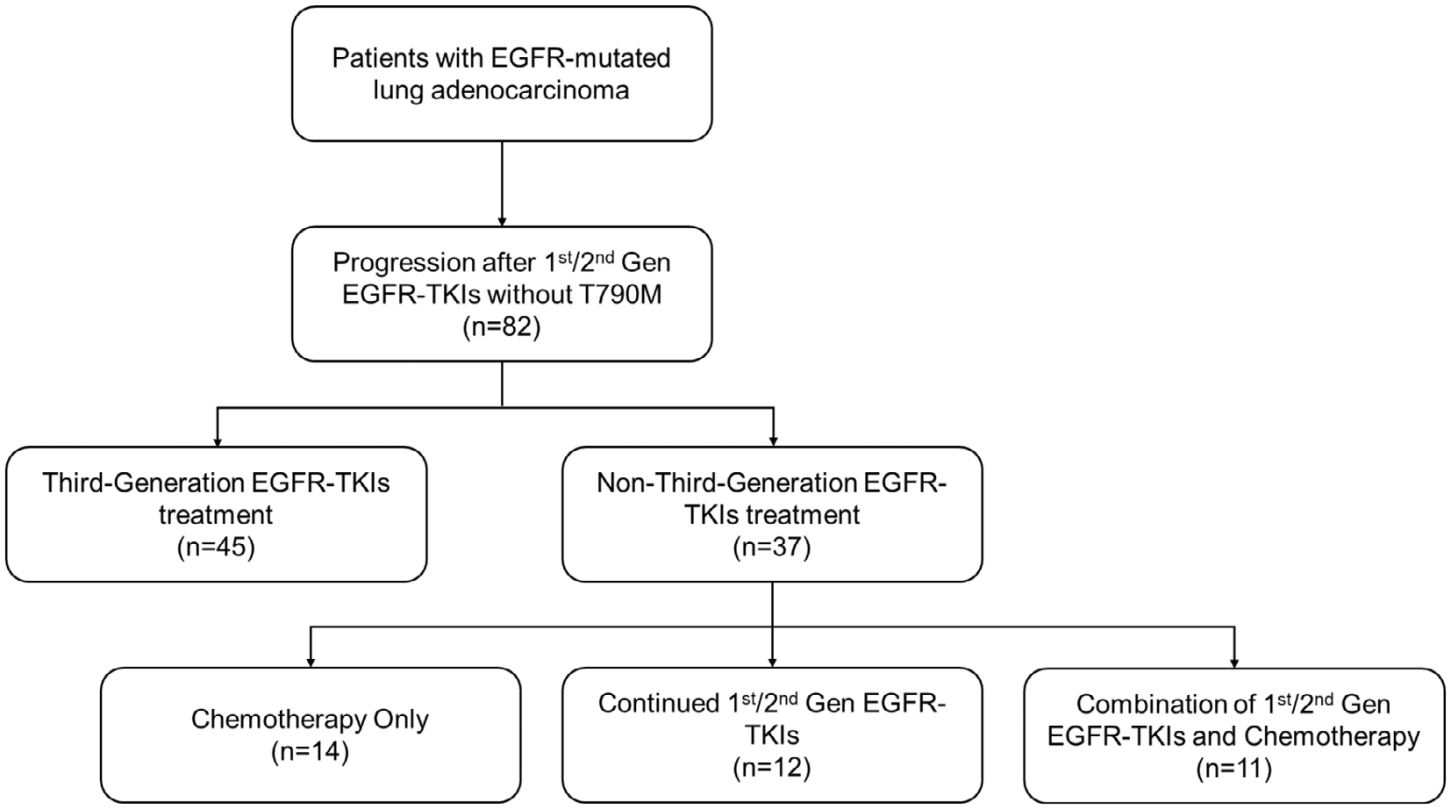
Flow chart of patient selection.

As of December 30, 2024, a total of 82 patients with advanced EGFR‐mutated lung adenocarcinoma were enrolled. EGFR mutations were tested by PCR, gene panel, or second sequencing, which were predominantly exon 19 deletion or L858R mutation. All patients progressed without acquiring T790M mutation after receiving first‐line treatment with first‐ or second‐generation EGFR‐TKIs, in which 45 patients received third‐generation EGFR‐TKIs and 37 received chemotherapy and/or first/s‐generation EGFR‐TKIs after progression (Figure 1). Baseline characteristics are summarized in Table 1. The majority of patients were non‐smoking females aged between 50 and 60, and the first‐line treatment primarily involved Gefitinib or Icotinib.

**TABLE 1:** Baseline characteristics of patients.

Characteristic	Total	3rd EGFR‐TKI	Non‐3rd EGFR‐TKI	*p* (3rd vs. non‐3rd)
	*N* = 82 (100.0%)	*N* = 45 (54.9%)	*N* = 37 (45.1%)	
Age	59.29 ± 9.53	58.82 ± 9.85	59.86 ± 9.23	0.625
BMI	20.33 ± 2.68	20.27 ± 2.87	20.40 ± 2.45	0.824
Sex				0.118
Male	32 (39.0%)	14 (31.1%)	18 (48.6%)	
Female	50 (61.0%)	31 (68.9%)	19 (51.4%)	
Smoke history				0.365
Never‐smoker	69 (84.1%)	36 (80.0%)	33 (89.2%)	
Smoker	13 (15.9%)	9 (20.0%)	4 (10.8%)	
Family history				0.038
Yes	4 (4.9%)	0 (0.0%)	4 (10.8%)	
No	78 (95.1%)	45 (100.0%)	33 (89.2%)	
ECOG status				0.686
ECOG 1	70 (92.1%)	38 (90.5%)	32 (94.1%)	
ECOG 2	6 (7.9%)	4 (9.5%)	2 (5.9%)	
Primary lesion				0.044
Left	32 (39.0%)	13 (28.9%)	19 (51.4%)	
Right	50 (61.0%)	32 (71.1%)	18 (48.6%)	
Gene Mutation				0.504
Exon 19	36 (45.6%)	18 (41.9%)	18 (50.0%)	
Exon 21	43 (54.4%)	25 (58.1%)	18 (50.0%)	
Disease stage				0.086
Stage III	6 (7.3%)	1 (2.2%)	5 (13.5%)	
Stage IV	76 (92.7%)	44 (97.8%)	32 (86.5%)	

### 3.2 Progression‐Free Survival

**FIGURE 2:**
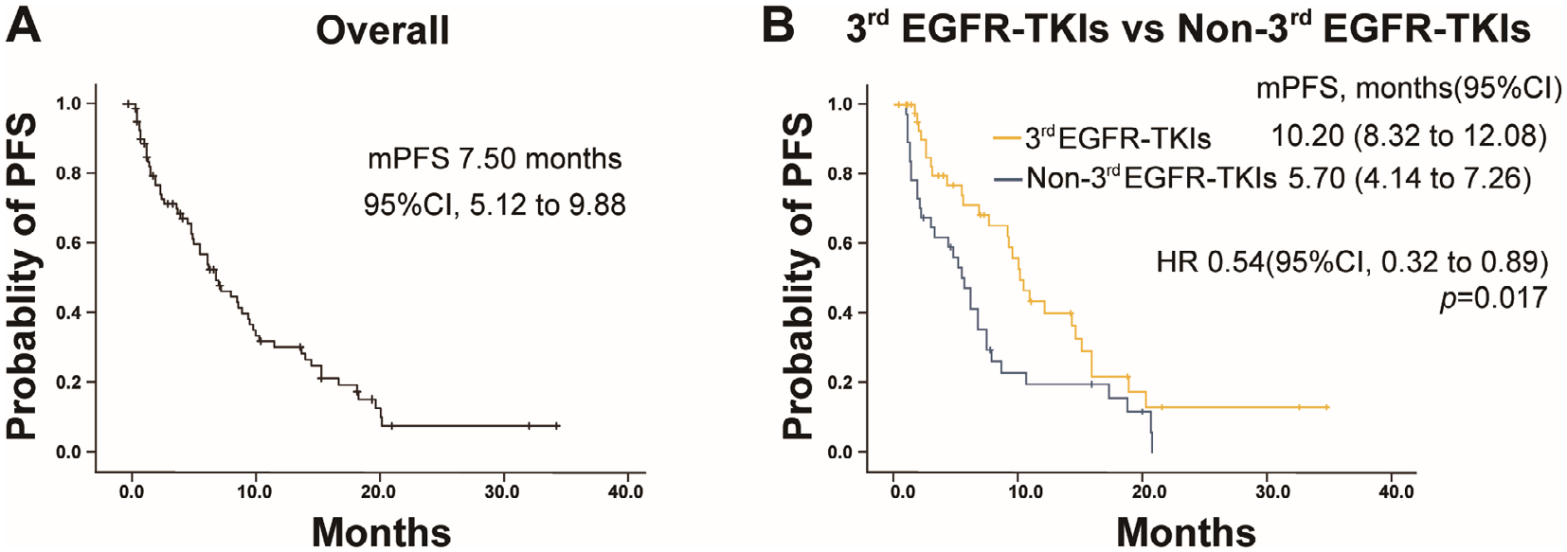
Kaplan–Meier plots of progression‐free survival (PFS). (A) PFS in the overall study population. (B) PFS in patients treated with third‐generation (3rd) EGFR‐TKIs versus non‐third‐generation (Non‐3rd) EGFR‐TKIs.

The median PFS for all patients (*n* = 82) was 7.50 months (95% CI: 5.12 to 9.88 months) (Figure 2A). The hazard ratio (HR) for PFS was 0.54 (95% CI: 0.32 to 0.89, *p* = 0.017), with a median PFS of 10.20 months (95% CI: 8.32 to 12.08) in the third‐generation EGFR‐TKIs group, compared to 5.70 months (95% CI: 4.14 to 7.26) in the non‐third‐generation EGFR‐TKIs group (Figure 2B).

**FIGURE 3:**
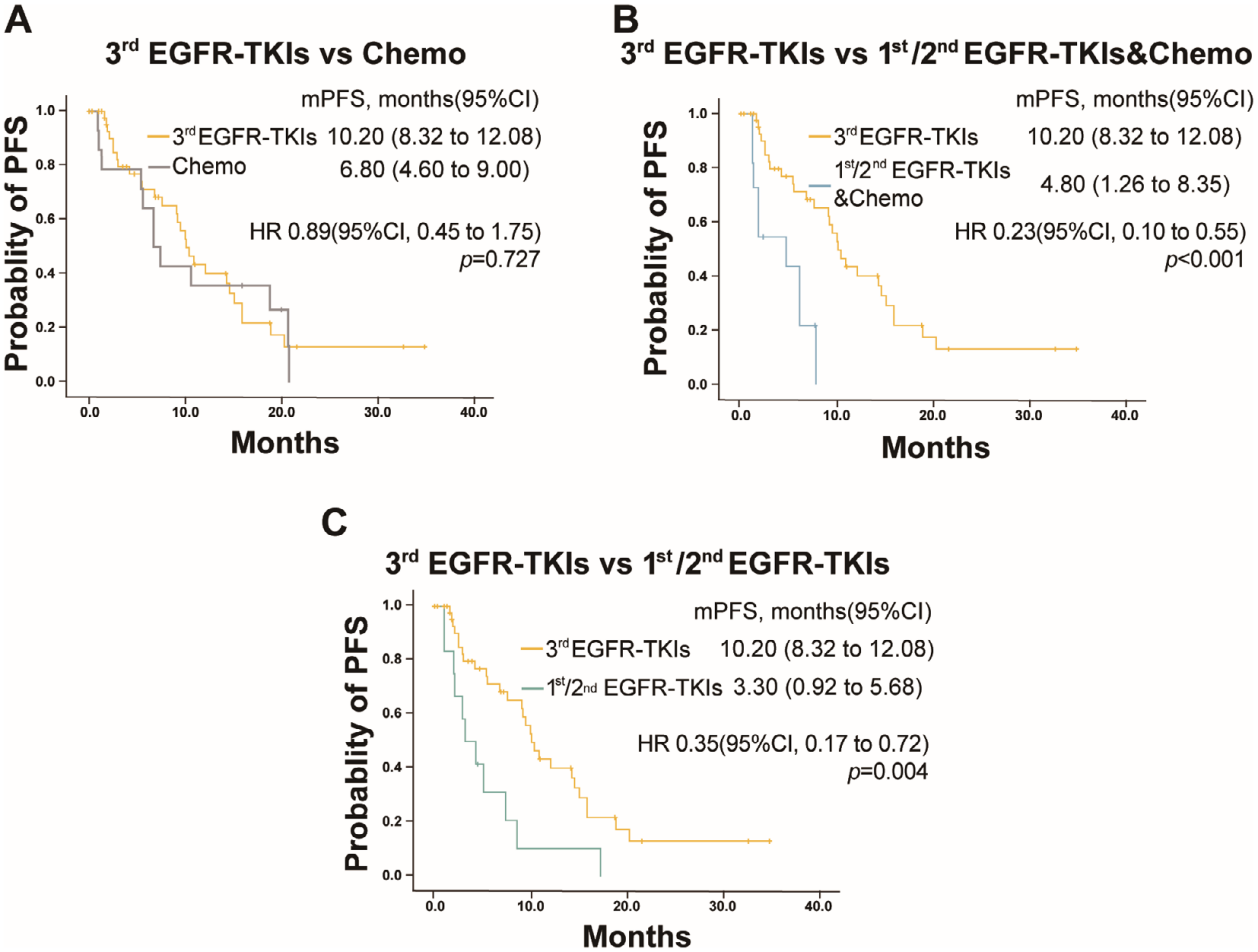
PFS in subgroup populations. (A) PFS in patients treated with third‐generation (3rd) EGFR‐TKIs versus chemotherapy (Chemo). (B) PFS in patients treated with third‐generation (3rd) EGFR‐TKIs versus first‐ or second‐generation (1st/2nd) EGFR‐TKIs combined with chemotherapy (Chemo). (C) PFS in patients treated with third‐generation (3rd) EGFR‐TKIs versus first‐ or second‐generation (1st/2nd) generation EGFR‐TKIs.

In the non‐third‐generation EGFR‐TKIs group, 14 out of 37 patients (37.8%) underwent chemotherapy after progression on first‐line targeted therapy. Twelve patients (32.4%) continued to receive first‐ or second‐generation targeted therapy (1st/2nd EGFR‐TKIs), while 11 patients (29.7%) were treated with a combination of first‐ or second‐generation EGFR‐TKIs and chemotherapy. Comparing the third‐generation EGFR‐TKIs group with the three subgroups, no significant difference in PFS was observed between the third‐generation EGFR‐TKIs group and the chemotherapy subgroup (10.20 months vs. 6.80 months, *p* = 0.727) (Figure 3A). However, the median PFS of the third‐generation EGFR‐TKIs group was significantly better than that of the 1st/2nd EGFR‐TKIs subgroup, whether combined with chemotherapy (10.20 months vs. 4.80 months, *p* < 0.001) or not (10.20 months vs. 3.30 months, *p* = 0.004) (Figure 3B,C).

**FIGURE 4:**
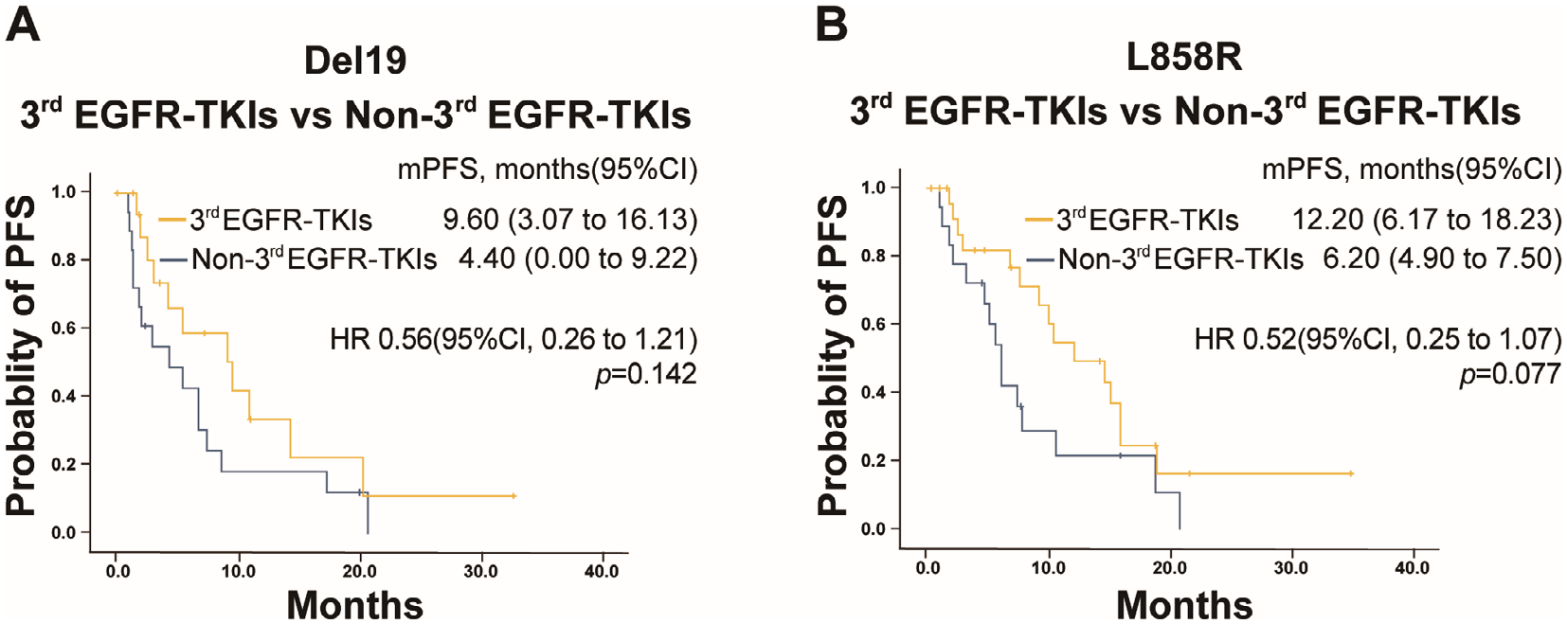
PFS in different EGFR mutation cohorts. (A) PFS in patients with EGFR exon 19 deletion (Del19) treated with third‐generation (3rd) EGFR‐TKIs versus non‐third‐generation (Non‐3rd) EGFR‐TKIs. (B) PFS in patients with L858R mutation treated with third‐generation (3rd) EGFR‐TKIs versus non‐third‐generation (Non‐3rd) EGFR‐TKIs.

In the mutation analysis, 36 (43.9%) and 43 (52.4%) of the 82 patients had exon 19 deletion and L858R mutation, respectively. The median PFS in the third‐generation EGFR‐TKIs group was longer than in the non‐third‐generation EGFR‐TKIs group for both exon 19 deletion (9.60 months vs. 4.40 months, *p* = 0.142) and L858R mutation (12.20 months vs. 6.20 months, *p* = 0.077), though the differences did not show statistical significance (Figure 4).

To further explore factors associated with progression‐free survival, we conducted both univariate and multivariate Cox regression analyses. In the univariate analysis, most baseline variables, including age, BMI, sex, smoking history, ECOG performance status, disease stage, and previous EGFR‐TKI therapy, were not significantly correlated with PFS (Table S1). Primary lesion site, however, was associated with increased risk of progression (HR 1.70, 95% CI 1.00–2.88, *p* = 0.049). In the multivariate analysis adjusting for potential confounders, primary lesion site remained an independent predictor of PFS (HR 1.77, 95% CI 1.03–3.05, *p* = 0.037), whereas other clinical variables showed no significant impact (Table S2). These findings suggest that primary lesion location may influence treatment outcomes, while other patient and disease characteristics had a limited effect on PFS in this cohort.

### 3.3 Overall Survival

**FIGURE 5:**
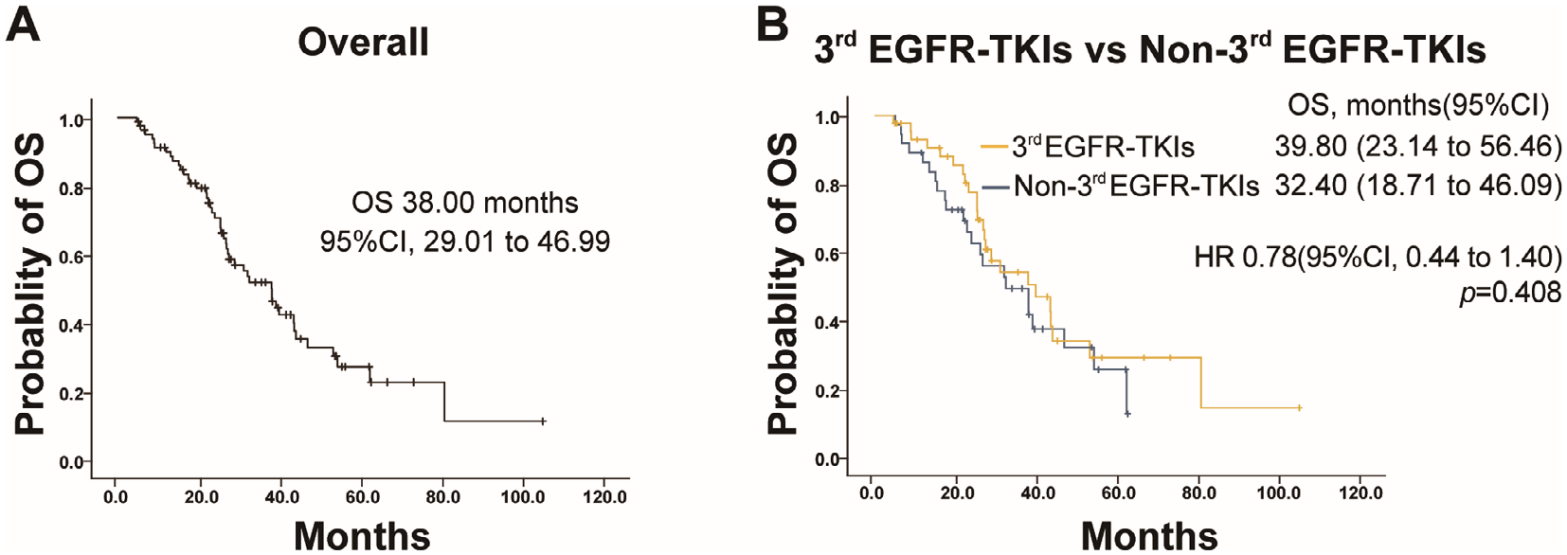
Kaplan–Meier plots of overall survival (OS). (A) OS in the overall study population. (B) OS in patients treated with third‐generation (3rd) EGFR‐TKIs versus non‐third‐generation (Non‐3rd) EGFR‐TKIs.

At the cutoff data, 47 out of 82 patients (57.3%) died, with 24 and 23 patients belonging to the third‐generation EGFR‐TKIs group and the non‐third‐generation EGFR‐TKIs group, respectively. The OS for all patients was 38.0 months (95% CI: 29.01 to 46.99 months), with a median OS of 39.80 months (95% CI: 23.14 to 56.46) in the third‐generation EGFR‐TKIs group and 32.40 months (95% CI: 18.71 to 46.09) in the non‐third‐generation EGFR‐TKIs group (Figure 5A,B). No statistically significant differences in OS were observed between the two groups (HR: 0.78, 95% CI: 0.44 to 1.40; *p* = 0.408) (Figure 5B). The estimated survival rates at 12, 24, and 36 months were 84.4%, 64.4%, and 33.3% in the third‐generation EGFR‐TKIs group, and 83.8%, 51.4%, and 37.8% in the non‐third‐generation EGFR‐TKIs group.

**FIGURE 6:**
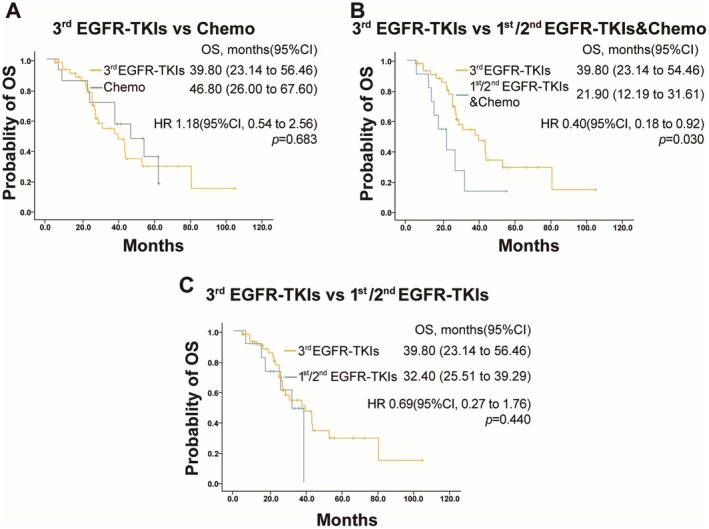
OS in subgroup populations. (A) OS in patients treated with third‐generation (3rd) EGFR‐TKIs versus chemotherapy (Chemo). (B) OS in patients treated with third‐generation (3rd) EGFR‐TKIs versus first‐ or second‐generation (1st/2nd) EGFR‐TKIs combined with chemotherapy (Chemo). (C) OS in patients treated with third‐generation (3rd) EGFR‐TKIs versus first‐ or second‐generation (1st/2nd) generation EGFR‐TKIs.

In the subgroup analysis, there were no significant differences in the OS between the third‐generation targeted therapy group and the chemotherapy subgroup (39.80 months vs. 46.80 months, *p* = 0.683) or the 1st/2nd EGFR‐TKIs subgroup (39.80 months vs. 32.40 months, *p* = 0.440) (Figure 6A,C). However, the OS in the third‐generation EGFR‐TKIs group was significantly better than that in the subgroup subjected to 1st/2nd EGFR‐TKIs combined with chemotherapy (39.80 months vs. 21.90 months, *p* = 0.030) (Figure 6B).

**FIGURE 7:**
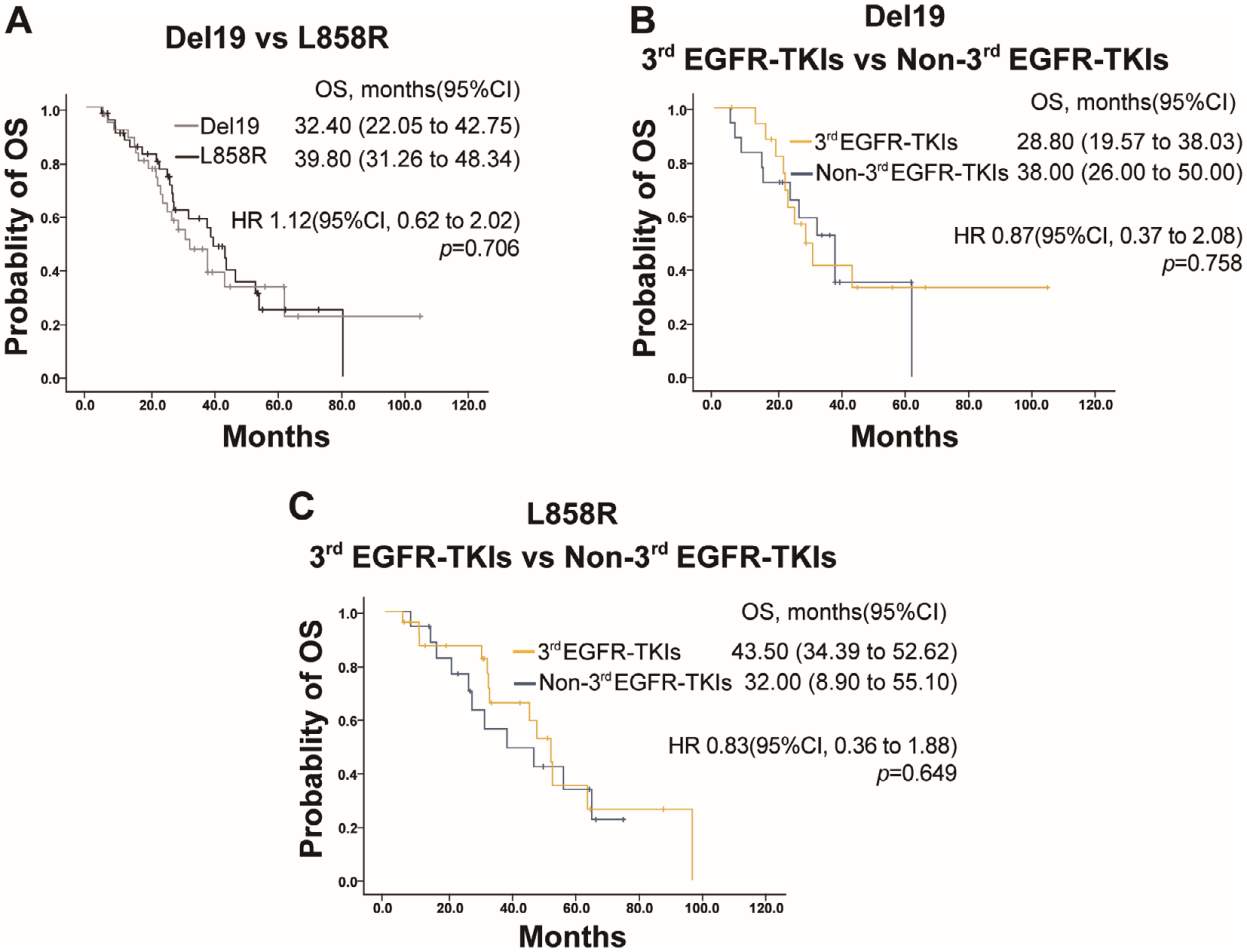
OS in different EGFR mutation cohorts. (A) OS in patients with EGFR exon 19 deletion (Del19) compared to those with L858R mutation. (B) OS in patients with EGFR exon 19 deletion (Del19) treated with third‐generation (3rd) EGFR‐TKIs versus non–third‐generation (Non‐3rd) EGFR‐TKIs. (C) OS in patients with L858R mutation treated with third‐generation (3rd) EGFR‐TKIs versus non–third‐generation (Non‐3rd) EGFR‐TKIs.

The median OS for patients with exon 19 deletion and L858R mutation was 32.40 and 39.80 months, respectively, with an HR of 1.120 (95% CI: 0.62 to 2.02, *p* = 0.706) (Figure 7A). The median OS in the third‐generation EGFR‐TKIs group and the non‐third‐generation EGFR‐TKIs group was 28.8 and 38.0 months in the exon 19 deletion subgroup (*p* = 0.758), and 43.5 and 32.0 months in the L858R subgroup (*p* = 0.649) (Figure 7B,C).

Patients with a good Eastern Cooperative Oncology Group (ECOG) performance status exhibited significantly longer survival, with an HR of 1.62 (95% CI: 1.02–2.55, *p* = 0.039) (Table 2). However, no significant differences were observed in OS analysis for other predefined subgroups, including age, body mass index (BMI), sex, smoking history, and disease stage, between the third‐generation EGFR‐TKIs group and the non‐third‐generation EGFR‐TKIs group (Table 2). In the multivariate Cox regression analysis, primary lesion site (HR 4.05, 95% CI: 2.12–7.72, *p* < 0.001) and ECOG performance status (HR 1.85, 95% CI: 1.04–3.28, *p* = 0.038) remained independent prognostic factors for OS, whereas other clinical characteristics, including age, BMI, smoking history, disease stage, family history, and previous EGFR‐TKI therapy, were not significantly associated with survival (Table 3).

**TABLE 2:** Univariate analysis of risk factors for overall survival.

Characteristic	Hazard ratio	*p*
Age	1.01 (0.98–1.04)	0.690
BMI	1.01 (0.91–1.12)	0.919
Sex	0.69 (0.37–1.29)	0.241
Smoke history	1.03 (0.43–2.43)	0.955
Family history	1.63 (0.50–5.30)	0.420
ECOG status	1.62 (1.02–2.55)	0.039
EGFR mutation at first diagnosis	1.21 (0.77–1.91)	0.414
Disease stage	4.26 (0.59–30.97)	0.153
Previous EGFR‐TKI therapy	1.14 (0.85–1.53)	0.372
Primary lesion	3.48 (1.89–6.40)	< 0.001
Past history	1.30 (0.73–2.32)	0.378

**TABLE 3:** Multivariate analysis of risk factors for overall survival.

Characteristic	Hazard ratio	*p*
Age	0.99 (0.95–1.02)	0.468
BMI	1.02 (0.91–1.14)	0.766
Smoking history	0.80 (0.33–1.92)	0.613
ECOG status	1.85 (1.04–3.28)	0.038
Disease stage	5.68 (0.77–41.87)	0.089
Previous EGFR‐TKI therapy	1.13 (0.82–1.56)	0.459
Primary lesion	4.05 (2.12–7.72)	< 0.001
Family history	0.666 (0.17–2.56)	0.554
Age	0.99 (0.95–1.02)	0.468
BMI	1.02 (0.91–1.14)	0.766
Smoking history	0.80 (0.33–1.92)	0.613

### 3.4 Safety

By the end of the study (December 30, 2024), adverse events (AEs) related to anticancer treatment occurred in 45 patients (54.8%) in the third‐generation EGFR‐TKIs group and 37 patients (45.1%) in the non‐third‐generation EGFR‐TKIs group, most of which were grade 1–2 AEs.

The safety profile of third‐generation EGFR‐TKIs was consistent with the primary adverse events report (Table S3), which included rash and acne (1 patient, 2.2%), nausea (1 patient, 2.2%), bone marrow suppression (1 patient, 2.2%), diarrhea (1 patient, 2.2%), and oral ulcers (1 patient, 2.2%) in the third‐generation EGFR‐TKIs group. In comparison, the non‐third‐generation EGFR‐TKIs group reported liver damage (2 patients, 5.4%), kidney damage (1 patient, 2.7%), and bone marrow suppression (3 patients, 8.1%). There was no treatment‐related death in either the third‐generation EGFR‐TKIs group or the non‐third‐generation EGFR‐TKIs group.

## 4 Discussion

The purpose of our study was to compare the efficacy of third‐generation EGFR‐TKIs with chemotherapy or first/s‐generation EGFR‐TKIs in T790M‐negative patients. This analysis is crucial for informing clinical decisions when selecting the optimal therapy for T790M‐negative patients with EGFR‐mutated advanced NSCLC after first‐ or second‐generation EGFR‐TKIs treatments.

In our study, the mPFS was longer in the group treated with third‐generation EGFR‐TKIs compared to those treated with non‐third‐generation therapies. Our findings highlight the potential of third‐generation EGFR‐TKIs as a treatment option for T790M‐negative patients with EGFR‐mutated tumors who have progressed following first‐ or second‐generation EGFR‐TKIs. However, our findings should be interpreted with caution. As this was a retrospective study, the strength of evidence is inherently weaker than that of prospective randomized trials. Prospective clinical studies focusing specifically on T790M‐negative populations remain limited, largely due to the challenges in patient recruitment and ethical considerations of withholding chemotherapy.

From a mechanistic perspective, accumulating evidence suggests that some T790M‐negative tumors may still rely on EGFR signaling through alternative resistance mechanisms. “Bypass” resistance mechanisms play a pivotal role in resistance to early‐generation EGFR‐TKIs, particularly in patients lacking the T790M mutation [4]. These T790M‐negative tumors with activation of pathways, such as MET and ERBB2, may continue to depend on EGFR signaling for their survival and proliferation [11]. The higher potency of third‐generation EGFR‐TKIs in inhibiting EGFR kinase activity might provide clinical benefit in this subset of patients [12]. Nevertheless, comprehensive molecular profiling is required to better identify which T790M‐negative patients could benefit most. Future prospective studies integrating genomic and translational analyses will be essential to validate our observations and clarify the biological rationale.

Chemotherapy has traditionally been the standard second‐line approach for patients with resistance to targeted therapies. The ORIENT‐31 study showed a significant survival benefit when using a combination of Sintilimab, IBI305, and chemotherapy. This combined treatment approach significantly extended the mPFS to 7.2 months, compared to only 4.3 months for patients who receive standard chemotherapy alone [13]. Our study found no significant difference in mPFS and OS between chemotherapy and third‐generation EGFR‐TKIs. However, patient compliance, a critical factor in real‐world treatment efficacy, was a concern as some patients in our retrospective study missed scheduled chemotherapy sessions. The third‐generation EGFR‐TKIs are often preferred due to better patient adherence and fewer side effects, which further supports the practicality of third‐generation EGFR‐TKIs for patients with acquired resistance and the absence of T790M mutation.

In our study, although third‐generation EGFR‐TKIs significantly improved PFS, no statistically significant differences in OS were observed between treatment groups. Several factors may explain this finding. First, subsequent lines of therapy after disease progression were heterogeneous in the real‐world setting, including cross‐over between targeted therapy and chemotherapy, which may have diluted the OS difference. Second, retrospective design and limited sample size reduced the statistical power to detect OS benefit. Third, patient compliance and treatment adherence could influence long‐term survival outcomes more substantially than short‐term disease control. These results suggest that while third‐generation EGFR‐TKIs offer meaningful disease control in T790M‐negative patients, the survival benefit may also depend on treatment sequence, subsequent therapy, and patient selection.

One limitation of our study was that not all patients underwent comprehensive next‐generation sequencing post‐progression, potentially missing additional resistance‐related mutations. Multiple molecular genetic tests are crucial in clinical practice for treatment response, resistance monitoring, and subsequent treatment planning. Our research also indicated that T790M mutation emerged in some patients who were treated with chemotherapy combined with first‐ or second‐generation EGFR‐TKIs after progression, emphasizing the need for repeated molecular testing at each progression stage.

It has been reported that patients with EGFR mutations may respond to EGFR‐TKIs re‐stimulation following a period of cytotoxic chemotherapy, possibly due to dynamic changes in tumor clones and the selective reduction of resistant clones [14]. For patients developing T790M mutation post third‐generation EGFR‐TKIs treatment, no unified treatment protocol currently exists. Approaches may include combining chemotherapy with EGFR‐TKIs, evaluating experimental drugs for specific mutations, participation in clinical trials, and incorporating local treatments like radiation therapy, tailored to each patient's genetic profile and circumstances.

The complexity and heterogeneity of lung cancer pose significant challenges to the accuracy of genetic testing, including the detection of the T790M mutation. For instance, the AURA phase I clinical trial revealed that 31% of patients initially found negative for the T790M mutation in tissue biopsies were later identified as T790M‐positive when plasma was tested [15]. This discrepancy can arise from factors such as variable detection sensitivities, low tumor cell content, improper sample handling, or inherent tumor tissue heterogeneity [16]. Therefore, comprehensive genetic testing is vital for patients who develop resistance to targeted therapies to ensure that they receive the most appropriate treatment based on their unique genetic profiles.

In conclusion, more rigorous prospective studies are necessary to evaluate the efficacy of third‐generation EGFR‐TKIs in T790M‐negative patients with resistance following first‐ or second‐generation EGFR‐TKIs. Clinicians should be cautious when considering third‐generation EGFR‐TKIs monotherapies in cases of EGFR‐TKIs resistance and confirmed T790M negativity. Active investigation for bypass activation mechanisms, such as mutations in MET, HER2, and BRAF, is crucial. Co‐targeting these specific mutations along with EGFR‐TKIs may offer comprehensive treatment strategies [17, 18].

Given the high costs of large‐panel DNA sequencing, many patients receive testing only for the T790M single point mutation upon resistance progression. The findings made in this study provide theoretical support for subsequent treatment strategies for NSCLC patients exhibiting EGFR‐TKIs resistance, emphasizing the importance of comprehensive genetic profiling in therapeutic decision‐making.

## Author Contributions

**Zhen Cheng:** conceptualization, investigation, formal analysis, writing – original draft. **Jiali Dong:** conceptualization, investigation, formal analysis, writing – original draft. **Huihao Lu:** investigation, writing – original draft. **Chuhong Huang:** investigation, writing – review and editing. **Shujun Li:** investigation, writing – review and editing. **Yanming Lin:** investigation, validation. **Yuting Chen:** investigation. **Yongcun Wang:** investigation. **Yanli Mo:** investigation. **Zhixiong Yang:** investigation, writing – review and editing. **Wenmei Su:** investigation, supervision, writing – review and editing.

## Ethics Statement

This study was conducted in compliance with the principles of the Declaration of Helsinki and was approved by the Ethics Committees of the Affiliated Hospital of Guangdong Medical University (LY2024‐10‐013). All participants in the study have been given informed consent.

## Conflicts of Interest

The authors declare no conflicts of interest.

## Supporting information


**Table S1:** Univariate analysis of risk factors for PFS.

**Table S2:** Multivariate analysis of risk factors for PFS.

**Table S3:** Adverse events in the two groups.

## Data Availability

The data that support the findings of this study are available on request from the corresponding author. The data are not publicly available due to privacy or ethical restrictions.
