# Supplementary material for: Third‐Generation EGFR‐TKIs in T790M‐Negative NSCLC After First/Second‐Generation EGFR‐TKI Failure: A Retrospective Study
Source: Cancer Med. 2025 Dec 16;14(24):e71302. doi: 10.1002/cam4.71302 (PMC12706518; doi:10.1002/cam4.71302)
Supplement: Supplementary file 1 — Table S1: Univariate analysis of risk factors for PFS. [file CAM4-14-e71302-s002.docx]

Supplementary Table 1. Univariate analysis of risk factors for PFS

| Characteristic | Hazard ratio | P Value |
| --- | --- | --- |
| Age | 0.98 (0.95-1.01) | 0.116 |
| BMI | 0.95 (0.87-1.05) | 0.298 |
| Sex | 1.10 (0.66-1.85) | 0.711 |
| Smoking history | 1.06 (0.55-2.06) | 0.855 |
| Family history | 2.34 (0.83-6.66) | 0.110 |
| EGFR mutation at first diagnosis | 1.32 (0.93-1.87) | 0.118 |
| ECOG status | 0.73 (0.43-1.25) | 0.250 |
| Disease stage | 0.83 (0.33-2.08) | 0.692 |
| Previous EGFR-TKI therapy | 1.16 (0.91-1.47) | 0.237 |
| Primary lesion | 1.70 (1.00-2.88) | 0.049 |
| Past history | 0.99 (0.60-1.65) | 0.970 |
