# Supplementary material for: Third‐Generation EGFR‐TKIs in T790M‐Negative NSCLC After First/Second‐Generation EGFR‐TKI Failure: A Retrospective Study
Source: Cancer Med. 2025 Dec 16;14(24):e71302. doi: 10.1002/cam4.71302 (PMC12706518; doi:10.1002/cam4.71302)
Supplement: Supplementary file 2 — Table S2: Multivariate analysis of risk factors for PFS. [file CAM4-14-e71302-s003.docx]

Supplementary Table 2. Multivariate analysis of risk factors for PFS

| Characteristic | Hazard ratio | P Value |
| --- | --- | --- |
| Age | 0.97 (0.95-1.00) | 0.079 |
| BMI | 0.94 (0.85-1.03) | 0.187 |
| Smoking history | 1.07 (0.54-2.12) | 0.855 |
| Disease stage | 0.77 (0.30-1.97) | 0.588 |
| Previous EGFR-TKI therapy | 1.16 (0.90-1.50) | 0.240 |
| Primary lesion | 1.77 (1.03-3.05) | 0.037 |
| Age | 0.97 (0.95-1.00) | 0.079 |
| BMI | 0.94 (0.85-1.03) | 0.187 |
| Smoking history | 1.07 (0.54-2.12) | 0.855 |
| Disease stage | 0.77 (0.30-1.97) | 0.588 |
| Previous EGFR-TKI therapy | 1.16 (0.90-1.50) | 0.240 |
