# Supplementary material for: Third‐Generation EGFR‐TKIs in T790M‐Negative NSCLC After First/Second‐Generation EGFR‐TKI Failure: A Retrospective Study
Source: Cancer Med. 2025 Dec 16;14(24):e71302. doi: 10.1002/cam4.71302 (PMC12706518; doi:10.1002/cam4.71302)
Supplement: Supplementary file 3 — Table S3: Adverse events in the two groups. [file CAM4-14-e71302-s001.docx]

Supplementary Table 3. Adverse events in the two groups

| Adverse event | 3^rd^ EGFR-TKI (n=45) | Non-3^rd^ EGFR-TKI  (n=37) |
| --- | --- | --- |
| Rash/Acne | 1 (2.2%) | 0 |
| Diarrhea | 1 (2.2%) | 0 |
| Nausea | 1 (2.2%) | 0 |
| Oral ulcer | 1 (2.2%) | 0 |
| Bone marrow suppression | 1 (2.2%) | 3 (8.1%) |
| Liver function impairment | 0 | 2 (5.4%) |
| Renal function impairment | 0 | 1 (2.7%) |
| Any AE (all grades) | 5 (11.1%) | 6 (16.2%) |
| Treatment-related death | 0 | 0 |
